# Partially Resistant Avocado Rootstock Dusa^®^ Shows Prolonged Upregulation of *Nucleotide Binding-Leucine Rich Repeat* Genes in Response to *Phytophthora cinnamomi* Infection

**DOI:** 10.3389/fpls.2022.793644

**Published:** 2022-03-11

**Authors:** Alicia Fick, Velushka Swart, Robert Backer, Aureliano Bombarely, Juanita Engelbrecht, Noëlani van den Berg

**Affiliations:** ^1^Department of Biochemistry, Genetics and Microbiology, University of Pretoria, Pretoria, South Africa; ^2^Forestry and Agricultural Biotechnology Institute, University of Pretoria, Pretoria, South Africa; ^3^Instituto de Biología Molecular y Celular de Plantas, Consejo Superior de Investigaciones Científicas - Universitat Politècnica de València (IBMCP-CSIC-UPV), Valencia, Spain

**Keywords:** NLR, avocado (*Persea americana* Mill.), *Phytophthora*, NB-LRR, resistance gene, NLR expression, Phytophthora root rot

## Abstract

Avocado is an important agricultural food crop in many countries worldwide. *Phytophthora cinnamomi*, a hemibiotrophic oomycete, remains one of the most devastating pathogens within the avocado industry, as it is near impossible to eradicate from areas where the pathogen is present. A key aspect to Phytophthora root rot disease management is the use of avocado rootstocks partially resistant to *P. cinnamomi*, which demonstrates an increased immune response following infection. In plant species, Nucleotide binding-Leucine rich repeat (NLR) proteins form an integral part of pathogen recognition and Effector triggered immune responses (ETI). To date, a comprehensive set of *Persea americana NLR* genes have yet to be identified, though their discovery is crucial to understanding the molecular mechanisms underlying *P. americana-P. cinnamomi* interactions. In this study, a total of 161 *PaNLR* genes were identified in the *P. americana* West-Indian pure accession genome. These putative resistance genes were characterized using bioinformatic approaches and grouped into 13 distinct *PaNLR* gene clusters, with phylogenetic analysis revealing high sequence similarity within these clusters. Additionally, *PaNLR* expression levels were analyzed in both a partially resistant (Dusa®) and a susceptible (R0.12) avocado rootstock infected with *P. cinnamomi* using an RNA-sequencing approach. The results showed that the partially resistant rootstock has increased expression levels of 84 *PaNLRs* observed up to 24 h post-inoculation, while the susceptible rootstock only showed increased *PaNLR* expression during the first 6 h post-inoculation. Results of this study may indicate that the partially resistant avocado rootstock has a stronger, more prolonged ETI response which enables it to suppress *P. cinnamomi* growth and combat disease caused by this pathogen. Furthermore, the identification of *PaNLRs* may be used to develop resistant rootstock selection tools, which can be employed in the avocado industry to accelerate rootstock screening programs.

## Introduction

Avocados (*Persea americana* Mill.) are an agriculturally important crop in many countries, including South Africa, Spain, and Mexico (Bulagi et al., 2016; Vargas-Canales et al., 2020). The annual gross production value of avocados in South Africa increased by 14.2% in 2018–2019 to a total of R1.42 billion, when compared to 2017–2018. Phytophthora root rot, caused by the hemibiotrophic oomycete, *Phytophthora cinnamomi* Rands, remains the largest threat to the avocado industry, in countries where the pathogen is present (Hardham and Blackman, 2018). The pathogen infects the fine feeder roots of avocado trees, leading to decreased water and nutrient transportation between cells (Coffey, 1987). A decline in tree health is observed which ultimately leads to plant death. *Phytophthora cinnamomi* can survive in soils over long periods of time through the production of chlamydospores and oospores, thus limiting the number of effective control methods for Phytophthora root rot (Dobrowolski et al., 2008; Belisle et al., 2019). Phosphite trunk injections, use of partially resistant rootstocks and organic mulching practices are methods currently employed by the avocado industry to control *P. cinnamomi* (Giblin et al., 2005). However, research has shown that *P. cinnamomi* has the potential to become less sensitive toward phosphite trunk injections (Dobrowolski et al., 2008). Continued screening for *P. cinnamomi* resistant rootstocks is thus of utmost importance and can be accelerated when host-pathogen interactions are understood.

Plant immune responses influence host-pathogen interactions and involve a myriad of proteins which activate complex, multilayered signaling pathways in response to pathogen attack (Dangl and Jones, 2001; Naveed et al., 2020). These can be categorized into two main responses; the Pathogen associated molecular pattern (PAMP) triggered immune response (PTI) and the Effector triggered immune response (ETI; Davis and Hahlbrock, 1987; Jones and Dangl, 2006). The recognition of PAMPs by membrane-bound Pattern recognition receptors (PRRs) activate an innate immune response, which is lower in amplitude when compared to the ETI response, and forms part of the plant’s first line of defense against pathogens (Matzinger, 2007). Pathogens, in turn, produce effector proteins which are secreted into plant cells to interfere with this process. These effector molecules may then be recognized by intracellular proteins, such as Resistance (R) proteins, either directly or indirectly (Monteiro and Nishimura, 2018). Upon effector recognition, R proteins are activated and trigger ETI—a high amplitude, robust immune response. The primary mode of action of ETI is to activate localized cell death caused by the Hypersensitive response (HR), aimed at arresting pathogen growth (Cui et al., 2015).

Resistance proteins are classified into five diverse groups based on protein structure and domains (Bezerra-Neto et al., 2020). The largest group consists of proteins with Nucleotide binding and Leucine rich repeat domains (LLRs), referred to as Nucleotide binding-Leucine rich repeats (NLRs; McDowell and Woffenden, 2003). Other groups include Receptor-like proteins (RLPs), Receptor-like kinases (RLKs), and Transmembrane Coiled-coil proteins (TM-CCs). The NLR group can be further sub-divided into two classes, based on the NLR’s N-terminus domain. The first class has a Coiled-coil (CC) domain, while the second class has a Toll/interleukin-1 receptor (TIR) structure domain. These NLRs are termed CNLs and TNLs, respectively. The CNL class also includes NLR proteins with both a CC domain and a RPW8 domain (resistance to powdery mildew), termed CC_R_-NLRs or C_R_NLs (Zhong and Cheng, 2016). *CNLs* are more abundant in the genomes of tree species when compared to *TNLs*, although the opposite is seen in *Arabidopsis* (Neale et al., 2017). Certain angiosperm and conifer species also do not follow this pattern due to *TNL* duplications, resulting in increased *TNL*:*CNL* ratios. Tandem duplications and *NLR* gene family expansions may have increased fitness levels of tree species that need long-term defense strategies against pathogens (Tobias and Guest, 2014). As a result, these duplicated gene sequences are mostly found in gene clusters within plant genomes (Meyers et al., 2003). Head-to-head *NLR* genes may express proteins which interact to form homo– or heterodimers, often vital for proper NLR function (Liang et al., 2019). These NLR protein dimers greatly increase the pathogen recognition potential of different NLR protein complexes (Van Wersch and Li, 2019).

A few NLR proteins are constantly expressed at low basal levels which allow plants to “scan” for invading pathogens (Meyers et al., 2002). Most of the genes coding for NLR proteins, however, show differential expression patterns after pathogen attack. This is influenced by the species of pathogen, excreted effector proteins and the plant’s genotype (Christie et al., 2016; Andam et al., 2020). In *Eucalyptus grandis* challenged by *Leptocybe invasa* and *Chrysoporthe austroafricana*, 218 and 343 *NLR*s were differentially expressed, respectively (Christie et al., 2016). RGA1, a TNL protein in the tree species *Salix viminalis*, showed higher expression in the resistant host when compared to its susceptible counterpart after *Melamspora larici-epitea* infection (Martin et al., 2016). Higher RGA1 expression allows for earlier ETI activation which ultimately leads to enhanced disease resistance. The level and timing of *NLR* expression is crucial, as this ultimately governs whether a plant would be successful in countering pathogen attack (Umadevi and Anandaraj, 2017). Transgenic plants with higher *NLR* gene expression demonstrated increased resistance to plant pathogens, even when these plants were transformed with non-native *NLR* genes. Expression of *ZmNB25*, a *NLR* first identified in maize, increased the resistance levels of *Arabidopsis* and rice toward *Pseudomonas syringae* pv. *tomato* DC3000 and *Bipolaris maydis*, respectively (Xu et al., 2018). Understanding how the expression of *NLR*s change during pathogen infection, and subsequently influence disease resistance, is vital to understanding complex plant-pathogen interactions.

To date, 49 complete putative *NLR* genes have been identified in avocado using microarray and RNA-sequencing analysis (Van den Berg et al., 2018; Pérez-Torres et al., 2021). In the study done by Pérez-Torres et al. (2021), Hass avocado stems were infected with *Fusarium kuroshium*, which causes *Fusarium* dieback disease in avocado. However, only four *NLR* genes were differentially expressed after *F. kuroshium* infection. Additionally, only a single avocado *NLR* gene has been implicated in the defense against *P. cinnamomi* infection in an avocado rootstock (Van den Berg et al., 2018). This *NLR*, functionally annotated as *RPP13*-like *protein 4*, showed increased expression after *P. cinnamomi* infection in a partially resistant rootstock. The use of RNA-seq and microarray data to identify *NLR* genes is limited by the fact that these genes need to be expressed to enable detection and identification. Avocado *NLR* gene identification has further been hampered by the lack of a high-quality genome assembly. Three avocado genomes, the Mexican landrace cultivar (*Persea americana* var. *drymifolia*), the Hass fruiting cultivar (Rendón-Anaya et al., 2019) and the West-Indian pure accession (WI) rootstock (Avocado Genome Consortium, Article in preparation) have only recently been sequenced, providing an opportunity to identify significantly more *NLR* genes within the avocado genome.

The discovery of *NLR* genes within the avocado genome could provide novel insight into the interactions of this plant with various pathogens. The current study set out to identify avocado *NLR* genes using the available genome sequences, and subsequently assess their expression during *P. cinnamomi* infection of partially resistant and susceptible rootstocks. We identified 161 putative *PaNLR* genes in the WI rootstock avocado genome, based on amino acid sequences characteristic of conserved NLR domains. Furthermore, we analyzed the expression of the candidate *PaNLR* genes in a partially resistant and susceptible rootstock following *P. cinnamomi* inoculation using an RNA-seq approach. We found significantly higher expression levels of 84 *PaNLR* genes in the partially resistant rootstock when compared to the susceptible rootstock after *P. cinnamomi* inoculation. This knowledge may benefit future rootstock screening programs aimed at increasing resistance levels toward *P. cinnamomi*. The *PaNLR* gene sequences identified in this study serve as an invaluable resource which can be used to pinpoint proteins that play a role in defense responses against other avocado pathogens.

## Materials and Methods

### Putative *PaNLR* Gene Identification

The *P. americana* West-Indian pure accession genome was obtained from the Avocado Genome Consortium (Article in preparation). Gene and protein names assigned during genome annotation (Peame105C00g000000) were abbreviated to PC00g000000. The Hass fruiting cultivar (*P. americana* cv. Hass; GCA_008087245.1) and Mexican rootstock (*P. americana* var. *drymifolia*; GCA_008033785.1) genomes (Rendón-Anaya et al., 2019) were obtained from GenBank (NCBI Genbank). Putative *Resistance* genes were identified and classified using the Resistance Gene Analog (RGA) prediction pipeline, RGAugury^1^ (Li et al., 2016; downloaded in September 2020). The avocado WI genome, as well as whole genome protein sequences from the WI, Mexican and Hass genomes were used as input with default parameters. The pipeline identifies conserved RGA sequences and domains using five programs: BLAST v. 2.10.1 (Camacho et al., 2009), nCoil^2^ v. 2.2 (Lupas et al., 1991), InterProScan^3^ v. 5.52-86.0 (Zdobnov and Apweiler, 2001), Pfam_scan^4^ v. 1.6 (Finn et al., 2010), and Phobius^5^ v. 1.01 (Käll et al., 2004). Putative NLR proteins were classified based on the identified domains, namely Nucleotide binding site (NB), Coiled-coil domain (CC), Coiled-coil with RPW8 domain (CC_R_), Toll/interleukin-1 receptor (TIR), and Leucine rich repeat domain (LRR). Here, N, C, T, and L represent NB, CC, TIR, and LRR domains, respectively. Thus, a protein classified as CNL has a CC, NB, and LRR domain, and a CN protein only has a CC and NB domain. RLKs, RLPs, and TM-CC classifications were annotated if the protein sequences contained a transmembrane domain. After identification and classification, protein functional annotation was done by performing BLASTp analysis in the non-redundant NCBI database. Searches were performed using an expected threshold value of 0.00001, with only the top hit for each candidate *NLR* gene being considered. If no significant match could be identified, proteins were annotated as Disease resistance-like (DRL) proteins.

### *PaNLR* Gene Cluster Identification

Gene clusters were defined based on appropriate definitions from Meyers et al. (2003), Kohler et al. (2008), and Christie et al. (2016). A gene cluster was defined as: a genomic region which contained three or more *NLR* genes, with less than nine other genes between adjacent *NLR* genes, and with two adjacent *NLR* genes being less than 250 kb apart. The WI genome general feature format (GFF) file was used to indicate the distance and number of neighboring genes between *NLR* genes. The position of *NLR* genes were visualized using CViT^6^ v. 1.3 (Cannon and Cannon, 2011).

### Phylogenetic Analysis

Phylogenetic analysis was used to assess whether *PaNLR* genes from the same gene cluster have high sequence similarity. Phylogenetic analysis included 161 *P. americana* NB-domain protein sequences, 10 complete protein sequences from *Cinnamomum micranthum* f*. kanehirae* (RWR97694.1, RWR95032.1, RWR91786.1, RWR92004.1, RWR93015.1, RWR98067.1, RWR88343.1, RWR88103.1, RWR87020.1, and RWR85657.1; Chaw et al., 2019) and one complete protein sequence from *Solanum bulbocastanum* (Q7XBQ9.1; Song et al., 2003). This *S. bulbocastanum* sequence was used since no RGA2 sequences were identified in *C. micranthum f. kanehirae* (Chaw et al., 2019). Sequence alignment was performed using ClustalW v2.1 with default parameters in MEGA X (Thompson et al., 1994; Kumar et al., 2018). A maximum likelihood phylogenetic tree was produced using the Jones-Taylor-Thornton substitution model and 1,000 bootstrap replications.

### Plant Inoculation and RNA Sequencing

*PaNLR* expression data were obtained by dual RNA-sequencing of *P. americana* inoculated with *P. cinnamomi*. Roots from partially resistant (Dusa®) and susceptible (R0.12) rootstocks were inoculated by dipping in *P. cinnamomi* (isolate GKB4) zoospore suspension with a concentration of 1.4 × 10^5^ zoospores/ml. Thereafter, plantlets were replanted in a mixture of vermiculite and perlite (1:1 ratio) and roots were harvested after 6, 12, 24, and 120 h post-inoculation (hpi). Three biological replicates from three independent plants were harvested at each time point. For control samples, three plantlets per rootstock were mock-inoculated using sterile water and root samples were harvested at 24 hpi.

Root samples were flash frozen using liquid N_2_ and stored at −70°C. The samples were then powdered using an IKA® Tube Mill (IKA®, Staufen, DUE). Modified CTAB extractions were performed to extract total RNA (Chang et al., 1993). RNA extractions were purified using a Qiagen RNeasy clean up kit (Qiagen, Valecia, California, United States) following DNase I treatment (Fermentas Life Sciences, Hanover, United States). An Agilent 2100 Bioanalyzer (Agilent Technologies, Santa Clara, CA, United States) was used to measure RNA purity and quality. Samples were stored at −70°C before being sent to Novogene (Novogene Corporation Inc., Chula Vista, California, United States) for paired-end (250–300 bp insert cDNA library) sequencing using Illumina Hiseq 2500 with PE150 mode.

### Expression Analysis

Dual RNA-sequencing data was analyzed by the Avocado Research Program and used during this study (Article in preparation). In short, RNA-seq reads were trimmed and low-quality bases were removed using Trimmomatic v. 0.39 (Bolger et al., 2014). FASTQC v. 0.11.9 was used to confirm read quality and the resultant reports were summarized using MultiQC (Ewels et al., 2016). RNA-seq reads were aligned to the *P. americana* WI genome using HISAT v. 2.0.6 (Kim et al., 2015). Gene level transcript abundance was quantified using featureCounts v. 2.0.1 (Liao et al., 2014) during initial expression screens within RNA-seq libraries across all time-points (6, 12, 24, and 120 hpi) using the mock-inoculated or susceptible rootstock libraries as a reference. DESeq2 (Love et al., 2014) was used for the normalization and analysis of counts. Library data points for transcripts with fewer than 10 reads were removed, and transcripts without any read data overall were omitted from further analyses. Quantification data for *PaNLR* genes were extracted using R studio v. 1.4.1106 (RStudio Team, 2020) and gene IDs previously identified by RGAugury. Expression level differences were analyzed using two approaches: (1) comparing the expression of candidate *PaNLR* genes 6, 12, 24, and 120 hpi in both the susceptible and partially resistant rootstock to that of their respective mock-inoculated samples, and (2) comparing the expression of candidate *PaNLR*s in the partially resistant rootstock to the expression in the susceptible avocado rootstock (mock-inoculated, 6, 12, 24, and 120 hpi). *PaNLR* genes were considered to be up- or downregulated when the Log_2_ Fold Change (Log_2_FC) value for each gene was ≥1 or ≤−1, respectively. False discovery rate adjusted values of *p* ≤ 0.05 generated as part of the DeSeq2 package were used to indicate statistical significance. Heatmaps and dendrograms depicting expression level differences (Log_2_FC) were generated using the Pheatmap package v. 1.0.12 (Kolde, 2012) in R studio v. 1.4.1106 (RStudio Team, 2020). To assess whether *NLR* genes within a gene cluster were co-expressed, *NLR* expression data was analyzed using Clust^7^ v. 1.12.0 (Abu-Jamous and Kelly, 2018).

## Results

### Putative *PaNLR* Genes Identified in the Avocado Genome

**Figure 1:**
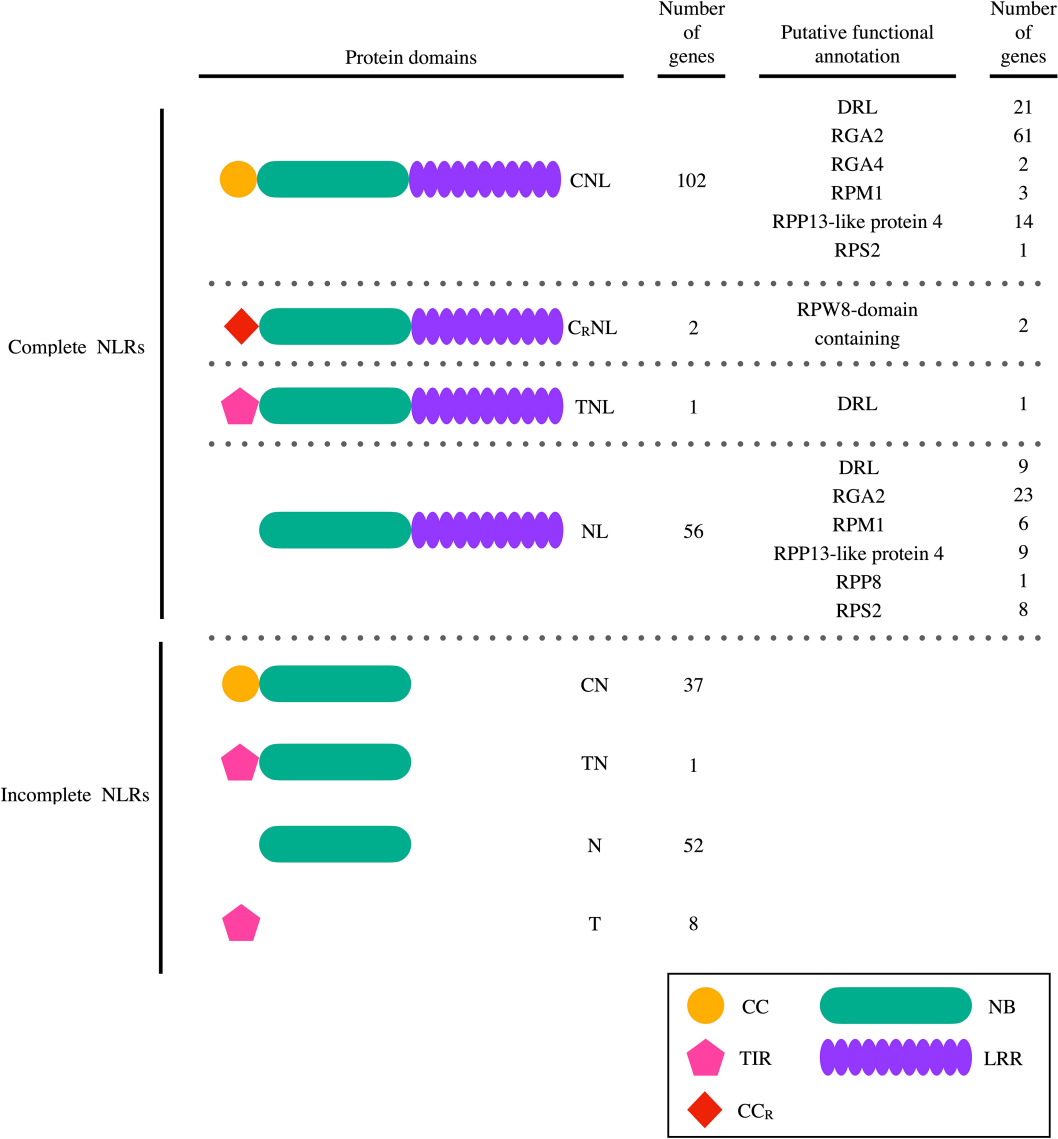
The number of *PaNLR* genes identified in the West-Indian pure accession *Persea americana* genome and the set of protein domains each gene encodes for. Putative Nucleotide binding-Leucine rich repeat (NLR) protein functional annotations predicted using BLASTp analysis are also listed (C/CC, coiled-coil domain; C_R_/CC_R_, coiled-coil RPW8 domain; DRL, disease resistance-like protein; L/LRR, leucine rich repeat domain; N/NB, nucleotide binding domain; and T/TIR, toll/interleukin-1 receptor domain).

The RGAugury pipeline identified 259 putative *PaNLR* genes within the WI rootstock genome (Figure 1), while no *PaNLR* genes could be identified within the Mexican and Hass genomes. *NLR* gene sequences which did not include a LRR domain sequence were removed from further analysis and considered as incomplete *PaNLR* genes. This resulted in 161 *PaNLR* sequences which were classified as complete *PaNLR* genes. Of these genes, 102 were classified as *CNLs*, two as *C_R_NLs*, 56 as *NLs* and one as *TNL*, based on the domains present within their predicted amino acid sequences.

Putative protein functional annotation of the 161 complete PaNLR candidates were assigned using BLASTp. In total, 31 sequences were assigned as DRL proteins. More than 52% of sequences were putatively identified as RGA2 proteins (Figure 1). Other sequence identifications included RGA4, RPM1, RPP13-like protein 4, RPP8, RPS2, and RPW8-domain containing type proteins.

The RGAugury pipeline also identified RLP, RLK, and TM-CC proteins using WI whole genome protein sequences. These protein sequences were separated from NLR sequences if a transmembrane domain sequence was identified. In total, 106 RLP sequences, 889 RLK sequences and 189 TM-CC sequences were identified.

### *PaNLR* Gene Clusters Identified in the WI Genome

**Figure 2:**
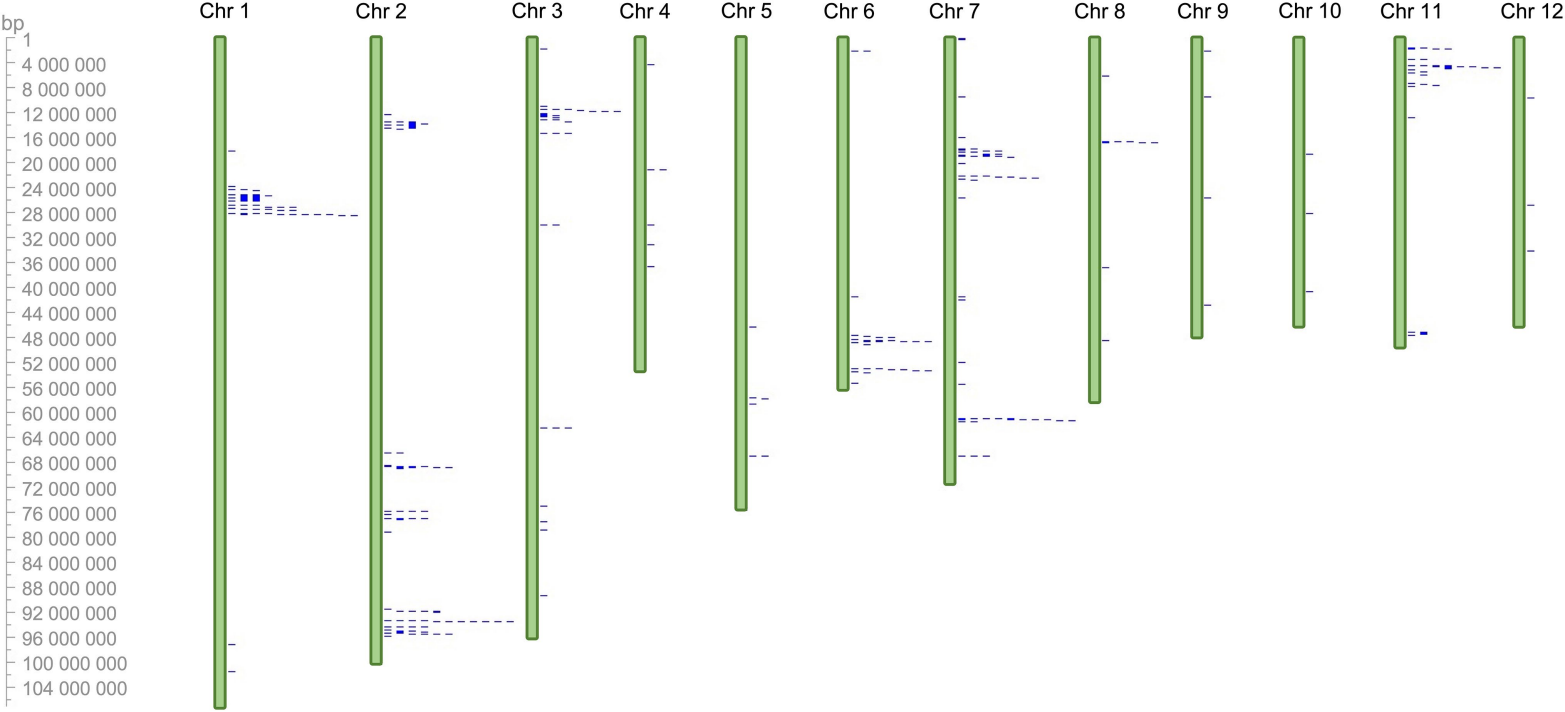
Chromosomal location of 148 putative *PaNLR* genes identified within the *Persea americana* West-Indian pure accession genome (represented by blue marks). The genes were mapped to 12 chromosomes (green bars) using CViT. Chromosome 0 was excluded from the analysis as it is not representative of a true chromosome, thus 13 *PaNLR* genes could not be mapped to chromosomes 1–12 and are not shown in the figure.

*PaNLR* gene clusters were identified based on neighboring *PaNLR* genes being less than 250 kb apart, and having less than three non-*NLR* genes between them. In total, 13 *PaNLR* gene clusters were identified, accounting for 74 (45.9%) of the complete *PaNLR* gene sequences (Figure 2). Thirteen *PaNLR* genes were mapped to unanchored chromosomes and were thus excluded from the cluster analysis. Chromosome 2 had four gene clusters (the largest set of clusters on any of the chromosomes) and also contained the largest gene cluster (consisting of nine *PaNLR* sequences). No gene clusters were identified on chromosomes 4, 5, 8, 9, 10, and 12. Eight of the gene clusters contained sequences which encode RGA2 proteins, with the gene clusters occurring on chromosomes 6 and 7 lacking *PaNLR* genes encoding RGA2 proteins (Table 1).

**Table 1:** Types of resistance genes found within *PaNLR* gene clusters on different chromosomes within the genome of *Persea americana* (West-Indian pure accession).

Chromosome	Cluster	Number of *PaNLR* genes	Type of PaNLR proteins encoded
1	1	7	RGA2
	2	8	RGA2
2	1	5	RGA2
	2	3	RGA2
	3	6	DRL, RGA2 and RGA4
	4	9	RGA2
3	1	3	RGA2
6	1	6	DRL
	2	5	RPS2, DRL and RPP13
7	1	4	RPM1 and DRL
	2	8	RPP13
11	1	3	RGA2
11	2	7	RGA2

### High *PaNLR* Sequences Similarity Within *NLR* Gene Clusters

**Figure 3:**
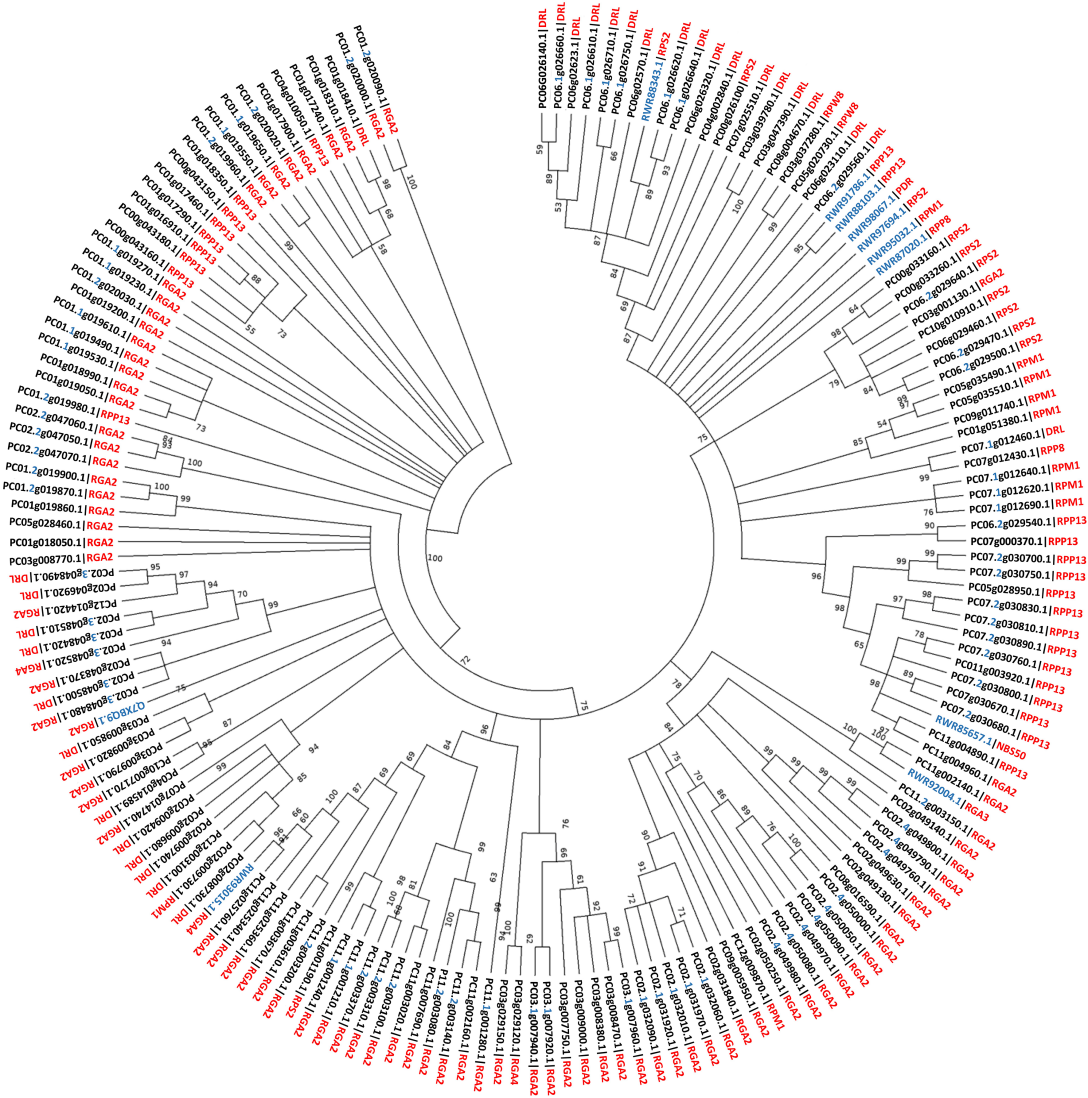
Phylogenetic relationship of 161 *Persea americana* (West-Indian pure accession) Nucleotide binding domains from putative *PaNLR* genes. Evolutionary history was inferred using the Maximum likelihood method and JTT matrix-based model following ClustalW alignment. A total of 1,000 bootstrap replicates were performed, with bootstrap values over 50 being shown above branch points. NB-domain protein sequences of *Persea americana* (PC) with complete NLR sequences from *Cinnamomum micranthum* f. *kanehirae* (RWR) and *Solanum bulbocastanum* (Q) were used during the analysis. *Persea americana* identification numbers include the gene cluster number, where appropriate (in blue) and protein type (in red). Unidentified PaNLR protein types were termed Disease resistance-like (DRL) proteins. Sequences from other species also include protein type (NBS50, NBS-LRR disease resistance protein NBS50; PDR, disease resistance-like protein isoform X1).

Phylogenetic analysis was performed to infer evolutionary relatedness between the 161 identified putative *PaNLR* genes using NB-domain protein sequences (Figure 3). Complete NLR protein sequences from *C. micranthum* f. *kanehirae* and *S. bulbocastanum* were included during analysis. The analysis revealed that *PaNLR* genes from the same *NLR* gene cluster grouped together within a clade, indicating high sequence similarity within clusters and possible gene duplication events. Most PaNLRs did not form a clade with *C. micranthum* f. *kanehirae* NLRs, indicating high diversification of *P. americana* NLRs after these two species diverged, especially RGA2 type PaNLRs.

### *PaNLR* Expression Following *Phytophthora cinnamomi* Inoculation

**Figure 4:**
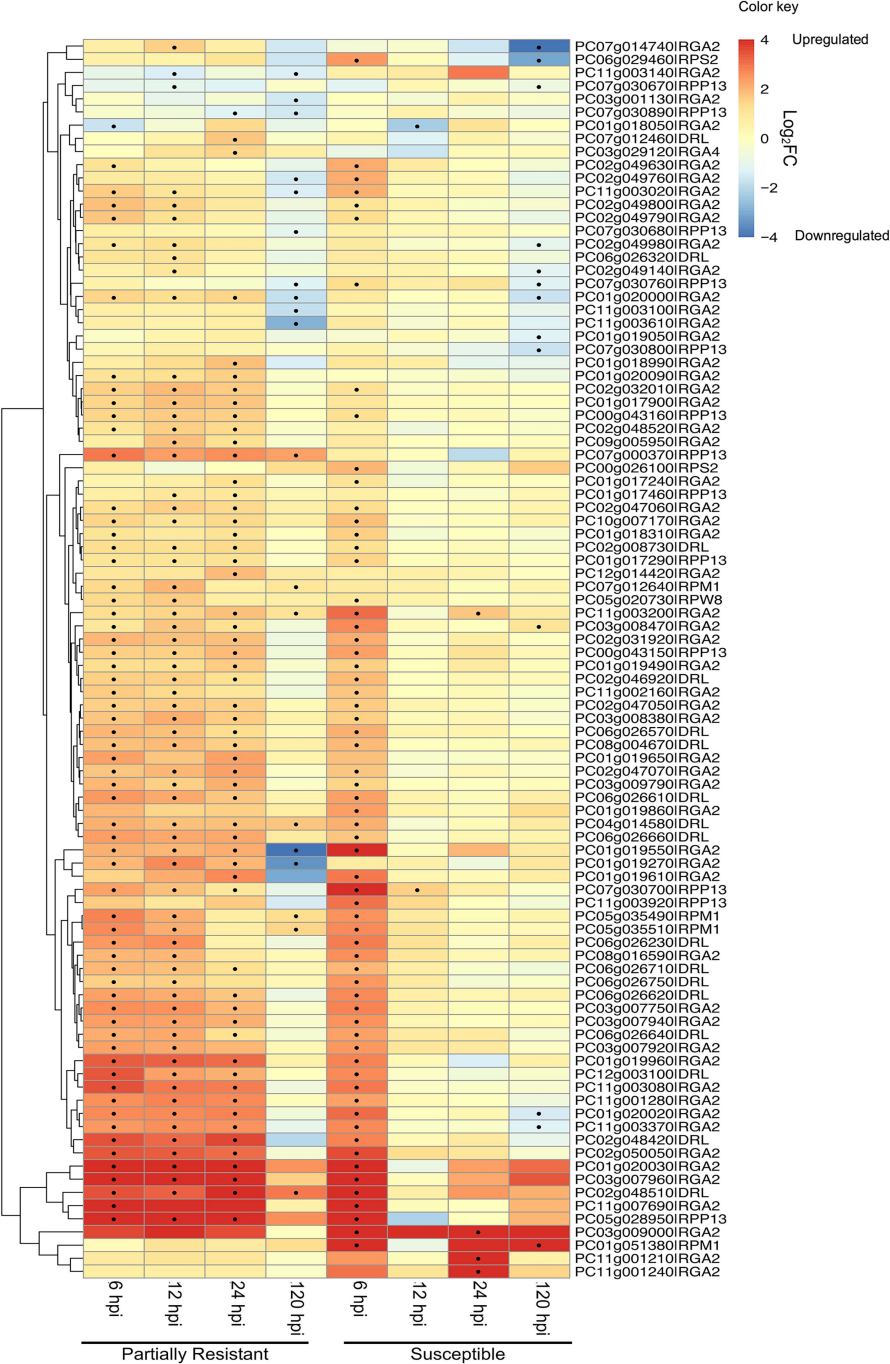
Heatmap and dendrogram showing the expression (as Log_2_ Fold Change) of 94 *PaNLR* genes following *Phytophthora cinnamomi* inoculation of a partially resistant (Dusa®) and susceptible (R0.12) avocado rootstock. Dots indicate a significant change (*p* ≤ 0.05 and |Log_2_FC| ≥ 1) in expression level when compared to mock-inoculated samples (hpi, hours post-inoculation).

Expression analysis was performed using dual RNA-sequencing data obtained from partially resistant and susceptible avocado rootstocks inoculated with *P. cinnamomi* zoospores. In total, 145 of the 161 identified complete *PaNLR* genes were expressed in the roots of both rootstocks, across all timepoints. A clear difference in *PaNLR* expression was observed between the two rootstocks in response to *P. cinnamomi* inoculation. In the partially resistant rootstocks (Dusa®), a total of 84 *PaNLR* genes showed a significant (*p* ≤ 0.05) change in expression level during at least one timepoint after *P. cinnamomi* inoculation, when compared to mock-inoculated samples (Figure 4). However, only 74 *PaNLRs* showed a significant (*p* ≤ 0.05) change in expression in the susceptible rootstocks (R0.12) after inoculation, when compared to mock-inoculated samples. The number of *PaNLR* genes with expression level differences in response to *P. cinnamomi* inoculation differed most notably between the two rootstocks at 12 and 24 hpi (Table 2). Only six *PaNLR* genes were differentially expressed in R0.12 at 12 and 24 hpi, compared to 74 *PaNLR* in Dusa®. *PaNLR* genes within a cluster were shown not to be co-expressed based on the Clust analysis.

**Table 2:** Number of *PaNLR* genes expressed in two avocado rootstocks in response to *Phytophthora cinnamomi* inoculation at different timepoints post-inoculation, when compared to mock-inoculated rootstocks (hpi, hours post-inoculation).

Time (hpi)	Partially resistant rootstock (Dusa®)		Susceptible rootstock (R0.12)		Common between rootstocks
	Upregulated genes	Downregulated genes	Upregulated genes	Downregulated genes	
6	63	1	64	1	54
12	64	2	1	1	1
24	55	2	4	0	2
120	7	12	2	11	2

PC03g007960|RGA2 was the most upregulated *PaNLR* gene in Dusa®, with a Log_2_FC value of 8.02 (*p* < 0.01) at 12 hpi (Figure 4). This gene was also upregulated in Dusa® at both 6 (Log_2_FC = 7.2; *p* < 0.01) and 24 hpi (Log_2_FC = 7.8; *p* < 0.01), while only being upregulated in R0.12 at 6 hpi (Log_2_FC = 7.8; *p* < 0.01). PC03g009000|RGA2 was the most upregulated *PaNLR* gene in R0.12 at 6 hpi with the largest Log_2_FC value of 8.46 (*p* < 0.01) of all samples. This gene did not show any significant changes in expression in any of the samples collected from Dusa®. Furthermore, PC11g001210|RGA2 and PC11g001240|RGA2 were upregulated in R0.12 at 24 hpi (Log_2_FC = 7.6; *p* < 0.01 and Log_2_FC = 5.1; *p* < 0.05, respectively), but did not show any significant change in expression in Dusa®, at any time point.

**Figure 5:**
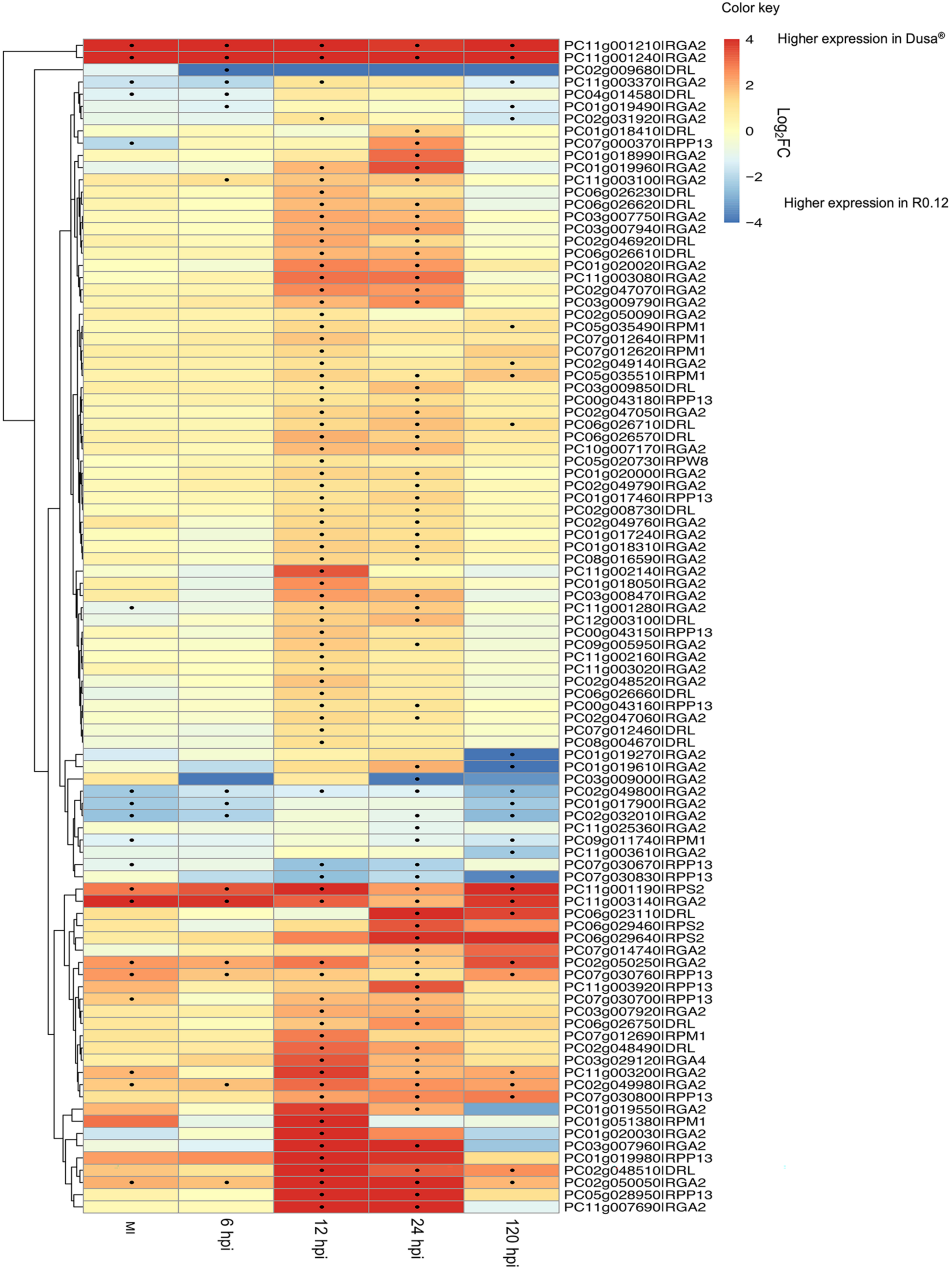
Heatmap and dendrogram showing *PaNLR* expression levels in a partially resistant avocado rootstock (Dusa®) mock-inoculated (MI) and following *Phytophthora cinnamomi* inoculation (hpi, hours post-inoculation) using a susceptible rootstock (R0.12) as the reference. A positive Log_2_FC indicates higher expression in the partially resistant rootstock, while a negative Log_2_FC indicates higher expression in the susceptible rootstock. Dots indicate a significant difference (*p* ≤ 0.05 and |Log_2_FC| ≥ 1) in expression between the two rootstocks.

When *PaNLR* gene expression was compared between the two rootstocks, with susceptible rootstock (R0.12) set as the reference, results indicated that *PaNLR* gene expression was higher in the partially resistant rootstocks (Dusa®), overall (Table 3). This was evident at the 12 and 24 hpi time points especially, with up to 74 *PaNLR* genes having higher expression (*p* ≤ 0.05) in Dusa® at 12 hpi (Figure 5). PC11g001210|RGA2 and PC11g001240|RGA2 were two *PaNLR* genes that were expressed at significantly higher levels in Dusa® when compared to R0.12, in all samples collected including mock-inoculated roots, even though both *PaNLRs* were significantly upregulated at 24 hpi in R0.12 when compared to mock-inoculated samples (Figure 4). The Log_2_FC values for both PC11g001210|RGA2 and PC11g001240|RGA2 were larger than 8.5 (*p* < 0.01) in all samples except at 24 hpi, where the Log_2_FC values decreased to 3.8 and 6.2 (*p* < 0.01), respectively (Figure 5). The *PaNLR* gene with the highest expression level in R0.12 when compared with Dusa®, was PC02g009680|DRL. However, this *PaNLR* was only expressed at significantly higher levels in R0.12 at 6 hpi (Log_2_FC = −7.6; *p* < 0.01), with no significant difference in expression levels being observed at any other time point.

**Table 3:** Number of *PaNLR* genes with a significantly higher expression in either the partially resistant (Dusa®) or susceptible (R0.12) avocado rootstock before and following *Phytophthora cinnamomi* inoculation (hpi, hours post-inoculation).

Time (hpi)	Genes with higher expression in Dusa®	Genes with higher expression in R0.12
mock-inoculated	10	9
6	9	7
12	74	3
24	61	7
120	16	11

## Discussion

Nucleotide binding-Leucine rich repeat proteins play a crucial role in plant immune responses by recognizing effector molecules produced by invading pathogens. Following effector recognition, NLR proteins activate ETI through complex signaling pathways, which leads to pathogen resistance (Monteiro and Nishimura, 2018). NLR proteins have been studied extensively in many other crops including *S. bulbocastanum*, *Zea mays*, *Oryza sativa*, and *Triticum monococcum* (Collins et al., 1998; Mago et al., 1999; Bozkurt et al., 2007; Lokossou et al., 2010). The results of these studies ultimately led to the breeding of crops with increased resistance toward various pathogens (Farnham and Baulcombe, 2006; Wang et al., 2019). Thus far, a comprehensive set of *P. americana NLR* genes have not been identified, and moreover, *P. americana NLR* gene expression has never been studied during *P. cinnamomi* infection. Thus, a large knowledge gap remains in understanding ETI activation during *P. cinnamomi* infection in avocado rootstocks (Van den Berg et al., 2018). The knowledge of avocado NLR functionality is vital to understanding resistance toward *P. cinnamomi* in avocado rootstocks and can be used by the avocado industry for molecular breeding purposes.

Using the *P. americana* WI genome, we identified 161 putative complete *PaNLR* gene sequences. No *PaNLR* gene sequences could be identified in the Mexican and Hass genome assemblies, since these genomes are highly fragmented (Rendón-Anaya et al., 2019; Talavera et al., 2019). Of the 161 complete *PaNLR* sequences, 102 were classified as CNL proteins, two as C_R_NL proteins, 56 as NL proteins, and one as a TNL protein (Figure 1). The 56 gene sequences encoding NL proteins were found to be expressed during *P. cinnamomi* infection, indicating that these proteins may play a role in this host-pathogen interaction, even though they lack CC and TIR domains. *NLR* sequences lacking these motifs are also expressed in other plant species, including *E. grandis*, *Malus* x *domestica*, and *Vitis vinifera*, further suggesting that these NLR proteins are still functional (Arya et al., 2014; Christie et al., 2016; Goyal et al., 2020). A higher *CNL*:*TNL* ratio was also observed in *E. grandis*, *M.* x *domestica*, and *V. vinifera* woody species, making it unsurprising to observe a higher *CNL*:*TNL* ratio in *P. americana*. However, it was not expected that only one *TNL* sequence would be identified. This could be a result of the genome assembly and annotation programs used not identifying full length gene sequences, thus producing truncated protein sequences as a result. BLASTp analysis was performed on the entire set of *P. americana* protein sequences and no additional *PaNLR* sequences could be identified. Furthermore, an independent study found only one *TNL* gene being expressed in Hass avocado stems during *F. kuroshium* infection (Pérez-Torres et al., 2021). This validated that no *TNL* motifs were missed during *PaNLR* identification using the RGAugury program and WI genome.

Putative protein functional annotation revealed that more than 50% of the identified *PaNLR* genes encode RGA2-like proteins (Figure 1). This type of NLR protein was first identified in *S. bulbocastanum* and is encoded for by *Rpi-blb1* (Van der Vossen et al., 2003). RGA2 proteins elicit an immune response and confer resistance toward *Phytophthora infestans* in potato and tomato plants, after recognizing ipiO RxLR proteins (Champouret et al., 2009). Recently, two *P. cinnamomi* RxLR proteins with high sequence similarity to *P. infestans* ipiO RxLRs were identified by Joubert et al. (2021). One of these RxLRs, PcinRxLR34a, was significantly upregulated in *P. cinnamomi* during infection of the susceptible rootstock R0.12, when compared to expression in mycelia. This suggests that this RxLR plays a role during pathogen infection. Future research should focus on identifying whether *P. americana* RGA2 proteins recognize these *P. cinnamomi* RxLRs.

RPP13-like protein 4 type proteins were the second largest group of PaNLR proteins identified in *P. americana*. RPP13-like protein 4 and RPP8 has been shown to confer resistance toward *Peronospora parasitica* and *Hyaloperonospora arabidopsidis*, respectively, in *Arabidopsis thaliana* (Bittner-Eddy et al., 1999; Mohr et al., 2010). *Peronospora parasitica*, *H. arabidopsidis*, and *P. cinnamomi* are oomycetes, suggesting that these pathogens may express Avirulence (Avr) proteins with similar structure and function (Cooke et al., 2000). This indicates that RPP13-like protein 4 and RPP8 in avocado may recognize *P. cinnamomi* effectors and play a role in rootstock resistance toward *P. cinnamomi*. The same assumption can be made regarding RPS2, which confers partial resistance toward *Phytophthora sojae*, a close relative to *P. cinnamomi*, in *Glycine max* (Mideros et al., 2007).

RPM1-like NLR proteins were also identified in avocado. Homologs of *RPM1*-like *NLR* genes in *A. thaliana* are responsible for recognizing *P. syringae* effectors during infection (Boyes et al., 1998). *Pseudomonas syringae* has been isolated from avocados; however, no symptoms of infection were observed (Scortichini et al., 2003). This might also explain why so few (5.6% of *NLRs*) *RPM1*-like genes were identified in avocado. Furthermore, since *P. syringae* infection does not present a threat to the avocado industry, *NLR* genes which confer resistance toward this pathogen would likely be of limited use in avocado screening programs. Lastly, two PaNLRs were annotated as RGA4-like proteins; in *O. sativa*, RGA4 proteins form heterodimers with RGA5 proteins, which recognize *Magnaporthe oryzae* infection (Césari et al., 2013). RGA5 proteins act as a receptor for *M. oryzae* Avr proteins and as a repressor of RGA4. Once RGA5 recognizes Avr proteins, RGA4 is released and activates cell death responses. Thus, in the absence of RGA5 proteins, RGA4 activates cell death in an Avr-independent manner (Césari et al., 2014). Since no *P. americana* proteins were identified as RGA5 proteins, it remains unclear whether the RGA4 proteins would respond to *P. cinnamomi* Avr proteins in avocado.

Gene cluster analysis was performed to identify possible duplication events of *P. americana NLRs* (Meyers et al., 2003). If *NLR* genes within a cluster were shown to be functionally important for rootstock resistance, *NLR* gene clusters can be targeted during molecular screening strategies. In total, 13 *PaNLR* gene clusters were identified in the *P. americana* genome (Table 1). Of these, four clusters were identified on chromosome 2 with one containing nine *PaNLR* gene sequences. No clusters were observed on chromosomes 4, 5, 8, 9, 10, and 12 (Figure 1). Eight clusters only contained RGA2 protein sequences, indicating that these genes may have originated from gene duplication events as described by Meyers et al. (1998) and López et al. (2003). Retained *NLRs* following duplication indicate functional relevance, suggesting that these RGA2 NLRs may play an important role in avocado defense responses. In *Phaseolus vulgaris*, *RGA2* gene clusters were identified as Quantitative trait loci (QTL), which confer resistance toward *Colletotrichum lagenarium* (López et al., 2003). Further investigation focusing on functional significance will help identify whether the *PaNLR* gene clusters in *P. americana* can be used as QTL molecular markers during rootstock breeding programs. Ultimately, these clusters serve as a reservoir for *NLR* diversity since duplicated genes are free to mutate, which may lead to novel NLRs being able to recognize novel effector proteins from pathogens (Innes et al., 2008).

Phylogenetic analysis revealed high similarity between *PaNLRs* within gene clusters, further indicating that *PaNLR* gene clusters may have originated from gene duplication events (Shao et al., 2014). During phylogenetic tree construction, 161 PaNLR Nucleotide binding domain protein sequences were used together with protein sequences from *C. micranthum* f. *kanehirae* and *S. bulbocastanum* (Song et al., 2003; Chaw et al., 2019). Sequences from *C. micranthum* f. *kanehirae* were used since this species is the closest relative to *P. americana* (both species form part of the Lauraceae family) in which NLRs have been identified (Wu et al., 2017). A RGA2 sequence from *S. bulbocastanum* was also included, since no RGA2 proteins were identified in *C. micranthum* f. *kanehirae* (Chaw et al., 2019). Phylogenetic analysis revealed that NB domain sequences within a *PaNLR* gene cluster grouped together, indicating high sequence similarity within these clusters (Figure 2). Moreover, few PaNLRs formed a clade with NLR sequences from *C. micranthum* f. *kanehirae*, indicating large *NLR* diversification within *P. americana* species. These observations might be the result of different pathogens shaping the *PaNLR* arsenal during the coevolutionary arms race between hosts and pathogens (Anderson et al., 2010).

Once putative *PaNLR* genes were identified in the WI genome, their expression was analyzed using dual transcriptomic data from partially resistant (Dusa®) and susceptible (R0.12) rootstocks inoculated with *P. cinnamomi*. Of the 161 *PaNLRs* identified in this study, 16 *PaNLRs* were not expressed in either rootstock at any timepoint. Many *NLRs* have tissue-specific expression levels in other plants, making these results unsurprising (Munch et al., 2018). Since this study investigated *PaNLR* expression in root tissues, it is expected that these 16 *PaNLRs* might play a role in recognizing pathogens which infect other avocado tissues. Interestingly, *PaNLR* genes within a gene cluster did not show similar expression patterns after *P. cinnamomi* inoculation. This was also observed in *E. grandis* when infected with *C. austroafricana* and *L. invasa*. The authors attributed this to expressed *NLR* genes being functionally relevant, and not the result of being located within active transcription zones by coincidence (Christie et al., 2016). Thus, we can hypothesize that *PaNLRs* in gene clusters being expressed following *P. cinnamomi* inoculation, do indeed have functional significance in activating defense responses against the invading pathogen.

During the first 6 h of infection, more than 60 *PaNLR* genes showed a significant increase in expression in either rootstock, with a similar pattern of expression activation for 54 of the same *PaNLR* genes in both rootstocks (Figure 4). This indicates that both rootstocks have similar responses with regards to *PaNLR* expression during the first 6 h of *P. cinnamomi* infection. *PaNLR* genes with the largest increase in expression at 6 hpi, were mainly RGA2 type proteins (PC01g020030, PC03g007960, and PC11g007690). RGA2 proteins activate the HR, and higher *RGA2* transcript levels were associated with increased *P. infestans* resistance in *S. bulbocastanum* (Bradeen et al., 2009). This upregulation of *RGA2* in both avocado rootstocks would likely result in a strong HR, which may limit *P. cinnamomi* growth.

*PaNLR* gene expression levels in Dusa® was higher when compared to R0.12, at both 12 and 24 hpi. Very few *PaNLR* genes showed differential expression patterns at 12 and 24 hpi in R0.12, which might indicate a decrease in ETI activation compared to Dusa®. Thus, the expression analysis revealed that Dusa® rootstocks overall have a stronger, more prolonged response to *P. cinnamomi* inoculation when compared to R0.12 rootstocks. *NLR* expression in susceptible varieties of *S. viminalis*, *C. arietinum* L. and *Brassica oleracea* do not show such stark differences in the expression when compared to resistant varieties, when infected with *Melampsora larici-epitea*, *Ascochyta rabiei*, and *Fusarium oxysporum* f. sp. *conglutinans*, respectively (Martin et al., 2016; Andam et al., 2020; Liu et al., 2020). It was thus expected that a greater portion of *PaNLRs* would show increased expression in R0.12 at these timepoints. These results might be due to either the pathogen interfering with *PaNLR* expression, or the pathogen suppressing host responses in R0.12. For example, W boxes, which are *cis*-regulatory elements recognized by WRKY transcriptions factors, are often overrepresented in plant defense-related gene promoters including *NLR* promoter sequences (Mohr et al., 2010). In *A. thaliana*, *WRKY* expression was downregulated by Avr3a-type effectors from *Phytophthora parasitica* (Li et al., 2019). This would subsequently lead to decreased *NLR* expression. It would be interesting to see whether *P. cinnamomi* uses similar tactics to influence ETI in *P. americana*. Thus, investigating which *cis*-regulatory elements are shared between *PaNLR* genes would be of interest in future research. Moreover, *P. cinnamomi* RxLRs were shown to have increased expression levels at 12 and 24 hpi in R0.12 (Joubert et al., 2021). Since some RxLRs suppress programed cell death, *P. cinnamomi* RxLRs could influence *PaNLR* expression and contribute to the results observed for R0.12 (Dalio et al., 2018). This data will help understand which PaNLR proteins might be important for recognizing *P. cinnamomi* effectors during infection and limiting *P. cinnamomi* growth. However, it must be noted that further studies, including protein–protein interaction studies, are needed to concretely state which individual PaNLR proteins recognize *P. cinnamomi* effectors.

A previous study, also done on R0.12 and Dusa® rootstocks, showed that R0.12 had significantly higher *P. cinnamomi* pathogen loads when compared to Dusa®, at all tested time-points (Engelbrecht et al., 2013). The increased *PaNLR* expression in Dusa®, especially *RGA2 PaNLRs*, at 12 and 24 hpi is likely to increase the amplitude of ETI activation and the HR, assuming successful *P. cinnamomi* Avr detection. Studies have shown that overexpression of *NLR* genes leads to higher levels of resistance and subsequent decreased disease symptoms. In *Nicotiana benthamiana* plants, overexpression of the *Vitis amurensis NLR* gene, *VaRGA1*, resulted in increased resistance toward *P. parasitica* (Li et al., 2017). Two *RGA2 NLRs* (PC11g001210 and PC11g001240) showed much higher expression in Dusa® when compared to R0.12, in all samples (Figure 5). As described earlier, the RxLR in *P. cinnamomi* with high similarity to a RGA2 protein counterpart, ipiO1, showed increased expression in R0.12 at 12 hpi, when compared to mycelia control samples (Joubert et al., 2021). Since R0.12 RGA2 proteins are not upregulated at this timepoint, it may suggest that fewer of these RxLR effectors are recognized, resulting in a compromised HR. However, high expression of *RGA2 NLRs* in Dusa® at all timepoints could result in increased ETI and might lead to decreased pathogen growth rates and/or decreased zoospore germination. However, in R0.12, pathogen load could be higher due to decreased ETI. These differences might be why Dusa® is able to survive *P. cinnamomi* attack for longer periods of time and show less disease symptoms.

This study is the first to identify and classify putative *PaNLR* genes using the *P. americana* WI genome. Phylogenetic analysis revealed that many *PaNLRs* found within *NLR* gene clusters may have originated from gene duplication events. Up to 94 *PaNLR* genes showed expression differences in response to *P. cinnamomi* attack, indicating a possible role in *P. cinnamomi* recognition and ETI activation. Furthermore, *PaNLRs* showed sustained, increased expression in a partially resistant rootstock (Dusa®) after inoculation, which could explain how this rootstock is able to suppress *P. cinnamomi* growth. This research paves the way toward understanding *P. americana*-*P. cinnamomi* interactions on a molecular level. Future studies should focus on investigating protein–protein interactions between PaNLRs and *P. cinnamomi* Avr proteins, and how *P. cinnamomi* is able to suppress *PaNLR* expression in R0.12 rootstocks. Furthermore, future studies should also include functionally characterizing the identified *PaNLRs* and investigating their role in defense responses against other *P. americana* pathogens. However, the lack of an efficient transformation system for *P. americana* greatly limits functional studies, and the results of this study highlights the need for the development of an improved system.

## Data Availability Statement

The datasets presented in this study can be found in online repositories. The names of the repository/repositories and accession number(s) can be found at: https://www.ncbi.nlm.nih.gov/genbank/, PRJNA675400.

## Author Contributions

AF analyzed all data and drafted the manuscript. RB performed the early analysis of RNA sequencing data and data curation. AB completed the assembly of the WI genome. JE designed and performed the experiments. VS and NB provided supervision of the study and revised the manuscript. All authors contributed to the article and approved the submitted version.

## Funding

Project funding was generously provided by the Hans Merensky Foundation.

## Conflict of Interest

The authors declare that the research was conducted in the absence of any commercial or financial relationships that could be construed as a potential conflict of interest.

## Publisher’s Note

All claims expressed in this article are solely those of the authors and do not necessarily represent those of their affiliated organizations, or those of the publisher, the editors and the reviewers. Any product that may be evaluated in this article, or claim that may be made by its manufacturer, is not guaranteed or endorsed by the publisher.

## Footnotes

^1^ https://bitbucket.org/yaanlpc/rgaugury/

^2^ https://bio.tools/ncoils

^3^ http://www.ebi.ac.uk/interpro/about/-interproscan/

^4^ http://pfam.xfam.org/

^5^ https://phobius.sbc.su.se

^6^ https://github.com/ekcannon/CViT

^7^ https://github.com/-BaselAbujamous/clust
